# Development of a dense SNP-based linkage map of an apple rootstock progeny using the *Malus* Infinium whole genome genotyping array

**DOI:** 10.1186/1471-2164-13-203

**Published:** 2012-05-25

**Authors:** Laima Antanaviciute, Felicidad Fernández-Fernández, Johannes Jansen, Elisa Banchi, Katherine M Evans, Roberto Viola, Riccardo Velasco, Jim M Dunwell, Michela Troggio, Daniel J Sargent

**Affiliations:** 1 East Malling Research (EMR), New Road, East Malling, Kent, ME19 6BJ, UK; 2 Biometris, Wageningen University and Research Centre, P.O. Box 100, 6700 AC, Wageningen, the Netherlands; 3 Istituto Agrario San Michele all’Adige, Via E. Mach 1, 38010, San Michele all'Adige, Italy; 4 WSU Tree Fruit Research & Extension Center, 1100 N. Western Ave., Wenatchee, WA, 98801-1230, USA; 5 School of Biological Sciences, University of Reading, Reading, RG6 6AH, UK

**Keywords:** Infinium, Golden Gate, Breeding, Selection, Genome sequence, Marker

## Abstract

**Background:**

A whole-genome genotyping array has previously been developed for *Malus* using SNP data from 28 *Malus* genotypes. This array offers the prospect of high throughput genotyping and linkage map development for any given *Malus* progeny. To test the applicability of the array for mapping in diverse *Malus* genotypes, we applied the array to the construction of a SNP-based linkage map of an apple rootstock progeny.

**Results:**

Of the 7,867 *Malus* SNP markers on the array, 1,823 (23.2%) were heterozygous in one of the two parents of the progeny, 1,007 (12.8%) were heterozygous in both parental genotypes, whilst just 2.8% of the 921 *Pyrus* SNPs were heterozygous. A linkage map spanning 1,282.2 cM was produced comprising 2,272 SNP markers, 306 SSR markers and the *S-*locus. The length of the M432 linkage map was increased by 52.7 cM with the addition of the SNP markers, whilst marker density increased from 3.8 cM/marker to 0.5 cM/marker. Just three regions in excess of 10 cM remain where no markers were mapped. We compared the positions of the mapped SNP markers on the M432 map with their predicted positions on the ‘Golden Delicious’ genome sequence. A total of 311 markers (13.7% of all mapped markers) mapped to positions that conflicted with their predicted positions on the ‘Golden Delicious’ pseudo-chromosomes, indicating the presence of paralogous genomic regions or mis-assignments of genome sequence contigs during the assembly and anchoring of the genome sequence.

**Conclusions:**

We incorporated data for the 2,272 SNP markers onto the map of the M432 progeny and have presented the most complete and saturated map of the full 17 linkage groups of *M. pumila* to date. The data were generated rapidly in a high-throughput semi-automated pipeline, permitting significant savings in time and cost over linkage map construction using microsatellites. The application of the array will permit linkage maps to be developed for QTL analyses in a cost-effective manner, and the identification of SNPs that have been assigned erroneous positions on the ‘Golden Delicious’ reference sequence will assist in the continued improvement of the genome sequence assembly for that variety.

## Background

The cultivated apple, *Malus pumila* Mill. (2*n* = 2*x* = 34), is a member of the Spireaoideae subfamily of Rosaceae, and is the fourth most economically-important fruit crop worldwide. As a consequence of the long juvenility phase of apple trees and of relatively high husbandry costs, the breeding and selection of novel apple rootstocks and scions is a time-consuming and costly procedure. Marker assisted selection (MAS) has the potential to increase the precision of apple breeding and an essential prerequisite to MAS is the production of high quality saturated genetic linkage maps to enable marker-trait associations to be made.

The genome of the apple scion cultivar ‘Golden Delicious’ was recently sequenced and assembled into 17 pseudo-chromosomes by an international consortium [1] using predominantly second generation 454 sequencing technology. Despite the inherent difficulties associated with contig assembly and gene-prediction in complex heterozygous genomes [2], the ‘Golden Delicious’ genome covers 598.3 Mbp, an estimated 71.2% of the *Malus* genome and almost complete coverage of the gene-space of the variety [1], with an average depth of sequencing 16.9×. Thus, the sequence provides a solid foundation for a wealth of downstream research activities including marker development, linkage map construction and marker-trait association.

The availability of data relating to single nucleotide polymorphisms (SNPs) from early in the development of the ‘Golden Delicious’ genome sequence meant that assays have been developed for screening segregating SNPs in apple mapping populations at relatively high throughput using the SNPlex and Golden Gate genotyping platforms [3,4], permitting not only the rapid development of linkage maps for *Malus* progenies, but the high resolution anchoring orientation of the scaffolded sequence data to a reference linkage map [1]. The degree of transferability of heterozygous SNPs between *Malus* varieties and species, and between *Malus* and *Pyrus* has recently been assessed [5], and estimates have been made about the number of SNPs required to allow the construction of a saturated linkage map in any given *Malus* progeny. In the study of Micheletti et al. [5], SNPs identified from the ‘Golden Delicious’ genome sequence were validated and tested for heterozygous transferability (T_SNP_) in a diverse selection of *Malus* germplasm. The investigation showed that SNPs identified within the ‘Golden Delicious’ sequence had an average transferability rate of 40.9% to *Malus* cultivars, with the lowest T_SNP_ to the cultivar ‘Wagner’ (25.7%) from those tested. The transferability rate in rootstock germplasm was between 29.9% and 39.7%, whilst to an accession of *Pyrus pyrifolia*, the T_SNP_ value was just 1.8%, demonstrating low cross-genera SNP transferability of the SNPs tested [5].

Through international collaboration, led by the RosBREED initiative in the USA [6], the *Malus* research community has developed an Infinium® II WGG genotyping array (referred to hereafter as the **I**nternational **R**osBREED **S**NP **C**onsortium array; IRSC array) for *Malus* and *Pyrus* using data from the re-sequencing of 27 *Malus* genotypes along with data from the ‘Golden Delicious’ genome sequence. The IRSC array contains a total of 7,867 *Malus* SNPs [7] in addition to 921 *Pyrus* SNPs. The development of this array represents a milestone in the development of molecular genetics and genomics resources for *Malus* and offers the promise of rapid, low-cost, high-throughput genotyping for the purposes of linkage map construction and the genotyping of germplasm collections, that will facilitate future QTL and genome-wide association studies. However, reports are yet to emerge of the efficacy of such arrays for genotyping germplasm and mapping populations from divergent sources or from species related to those for which they were originally designed.

The M432 mapping progeny [8,9] has been raised for the study of genes controlling traits of relevance to rootstock breeding, with the long-term aim of developing robust markers for MAS. Since the progeny is derived from parental rootstock varieties, the genetic basis of the seedlings that comprise the population represents a departure from the well-characterised scion genotypes that were used to identify SNPs for the construction of the IRSC array [7]. The progeny has been previously characterised with *S*-locus-specific markers, and 323 (306 co-dominant, and 17 dominant) SSR markers [9] distributed throughout the *Malus* genome. A comprehensive consensus linkage map of the progeny has been developed spanning 17 linkage groups (LGs), with an average marker density of one marker every 3.79 cM. The linkage map was partially anchored to the ‘Golden Delicious’ genome sequence, and a total of 47% of the sequence that was contained in metacontigs could be assigned positions on the M432 map.

The aim of this investigation was to test the IRSC array in the M432 rootstock mapping progeny to determine its utility to genome-wide saturated map construction. Additionally, we aimed to increase the percentage of the *Malus* genome sequence that could be directly related to regions of the M432 linkage map for the purposes of candidate gene identification and marker development following QTL analysis. We evaluated the SNP-based linkage map produced against the previously-published SSR-based linkage map of the population developed by Fernández-Fernández et al. [9]. We compared the positions of SNP markers on the M432 linkage map with their predicted positions on the ‘Golden Delicious’ genome sequence and assessed the accuracy of the genomic placement of the heterozygous SNP markers in relation to the genetic positions of the markers on the M432 map. An evaluation was made of the ease at which the IRSC array could be implemented in the mapping progeny in relation to previous SSR assays performed for linkage map construction in this progeny.

## Results

### SNP heterozygosity in the M432 mapping progeny

GenTrain scores for all SNPs scored in the M432 progeny ranged from 0.043 to 0.961, with an average of 0.723. Cluster separation ranged from 0.004 to 1 with an average of 0.814. Of the 8,788 SNP markers contained on the IRSC array (7,867 *Malus* SNPs and 921 *Pyrus* SNPs), 664 markers failed in both parental genotypes, 44 failed in the ‘M.27’ genotype and a further 51 markers failed in the ‘M.116’ genotype. A total of 5,078 markers were homozygous in both parental genotypes of the M432 progeny, and 95 revealed unexpected genotypes in the progeny given the parental genotypes. Of the remaining 2,856 markers 1,007 were heterozygous in both parents, 976 markers were heterozygous only in the ‘M.27’ parental genotype, and 873 markers were heterozygous only in ‘M.116’.

### SNP and SSR co-segregation analysis and linkage map construction

**Figure 1:**
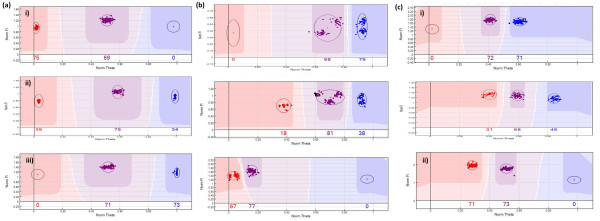
**SNP genotype clusters revealed following analysis by GenomeStudio.** Examples of SNP genotype clusters revealed following analysis using GenomeStudio generated from the M432 mapping progeny using the IRCS genoptying array. (**a**). Expected patterns of genotype clusters for markers with the parental genotype conformation i) AA × AB, ii) AB × AB and iii) AB × BB. (**b**). Genotype clusters displaying evidence of sub-clusters possibly as a result of hybridisation to paralogous loci. (**c**). Clusters not locating to the expected region of graph space, leading to possible mis-assignation of genotype in the i) AB × BB, and ii) AA × AB marker types.

GenTrain scores for the markers considered for mapping ranged from 0.4 to 0.961, with an average of 0.713. Cluster separation scores ranged from 0.347 to 1, with an average of 0.848. A total of 854 SNPs heterozygous in the ‘M.27’ parental genotype, 751 SNPs heterozygous in the ‘M.116’ parental genotype, and a further 665 SNPs heterozygous in both parental genotypes of the M432 progeny coalesced into the 17 expected LGs for a consensus genetic linkage map. Thus, a total of 586 putatively segregating loci were not located to LGs on the M432 map. Close visual inspection of these loci in GenomeStudio (Illumina) revealed that the majority had poor cluster separation scores and did not cluster as expected despite having high GenTrain scores. In many cases, the clusters that were produced were composed of a number of smaller sub-clusters and of individual genotype calls that did not cluster tightly or that did not cluster in the expected region of the graph space and were shifted from either the 0 or the 1 axis (Figure 1). As such, cluster patterns at these loci were either unsuitable for genotypic analysis due to mis-assignation of sub-clusters, or it was not possible to reliably determine the heterozygosity status of the parental genotypes, and thus the loci were not considered for further analyses.

**Figure 2:**
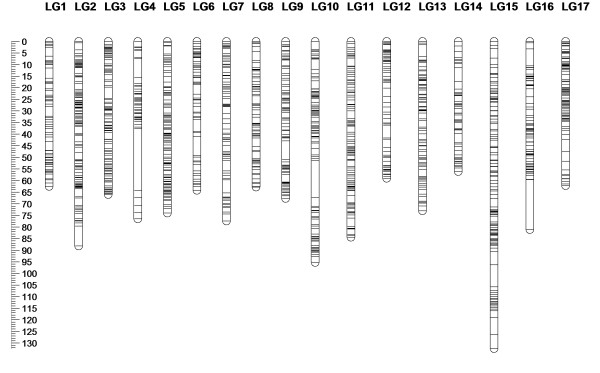
**SNP-based linkage map of the M432 progeny.** An consensus genetic linkage map of the M432 *Malus* mapping population composed of 2,579 molecular markers, including 2,272 SNPs generated with the IRSC array, 306 SSRs and the *S*-locus, spanning 1,282.2 cM over 17 LGs. The scale in centi-Morgans is given at the edge of the figure.

In addition to the 2272 SNPs, the 306 codominant SSR markers and the *S*-locus that were previously mapped by Fernández-Fernández et al. [9] were located to the 17 LGs. Previously mapped dominant SSR markers segregating AØ × AØ were not considered for mapping in this investigation. The consensus linkage map of the M432 progeny is presented in Figure 2 and Table 1 summarises the marker composition and lengths of the 17 M432 LGs. The map spanned a total of 1,282.2 cM and contained a total of 2,579 molecular markers. LG 14 was the shortest LG on the map, spanning 56.1 cM, whilst LG15 was the longest, spanning 132.4 cM. The highest number of markers mapped to a single LG was 221 on LG2; the smallest number of markers on a single LG was 69 on LG4. The map had an average marker density of one marker every 0.5 cM. The linkage map contained a total of three regions in excess of 10 cM that contained no mapped molecular markers.

**Table 1:** The number of markers and the genetic distances mapped on the M432 SNP-based linkage map

**Linkage group**	**Length**	**ABxAA SNP**	**AAxAB SNP**	**ABxAB SNP**	**SSR**	**Total markers**	**Segregation distortion**
**1**	62.459	24	43	42	14	123	0-3 cM; 12 markers
**2**	79.641	34	98	74	15	221	
**3**	66.027	75	53	54	14	196	31-76 cM; 13 markers
**4**	76.508	16	12	33	8	69	
**5**	81.756	73	44	52	23	192	
**6**	64.207	41	43	23	12	119	
**7**	77.391	40	31	43	15	129	0-5 cM; 6 markers
**8**	62.792	46	39	22	12	119	
**9**	67.72	46	40	47	25	158	7-57 cM; 36 markers
**10**	95.275	25	41	42	25	133	
**11**	84.42	66	63	43	28	200	
**12**	59.003	19	22	51	16	108	10-16 cM, 20–36 cM, 49 cM, 54–58 cM; 33 markers total
**13**	73.039	66	44	31	16	157	
**14**	56.136	82	18	22	13	135	
**15**	132.444	69	65	39	25	198	
**16**	81.134	52	66	21	20	159	
**17**	62.242	82	29	26	26*	163	31-61 cM; 13 markers
**Total**	**1282.194**	**854**	**751**	**665**	307*	**2579**	

The total number of SNP and SSR markers mapped in the M432 mapping progeny, the number of markers per chromosome and the total length of each LG in centi-Morgans (cM).

The largest region to which no markers were mapped was 26.6 cM on LG4 which was defined by four SSR markers that had been previously mapped by Fernández-Fernández et al. [9]. Similarly, a region at the distal end of LG16 spanning 26.5 cM containing no markers was defined by a single SSR marker previously mapped by Fernández-Fernández et al. [9]. Upon closer inspection, the segregation of these SSR loci were distorted towards an excess of an allele from ‘M.27’ parental genotype which could have potentially led to an overestimation of the genetic distance between these markers and their closest flanking markers along the LG. The markers were tentatively included on the linkage map presented since their position and order corresponded with the previously published M432 linkage map.

**Figure 3:**
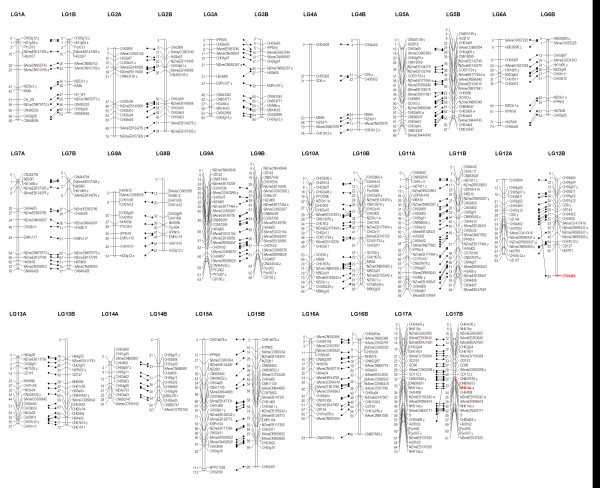
**Comparison of SSR positions on the M432 linkage map.** A comparison of the genetic positions of 306 SSR markers and the *S*-locus in the M432 mapping progeny. Marker positions on the left (**A**) were determined following mapping of only SSR markers by Fernández-Fernández et al. [9], whilst those on the right (**B**) were determined following mapping of an additional 2,269 SNP markers in this investigation. Marker order was generally conserved except for the positions of two loci (Ch05d03; LG12 and NH014a.z; LG17) given in red which were removed from subsequent analyses.

Figure 3 shows a comparison of the order of SSR markers in the M432 map between the current investigation and the report of Fernández-Fernández et al. [9]. Marker order of previously mapped SSRs in the M432 progeny was generally well conserved, with only minor changes compared to the previous SSR map of Fernández-Fernández et al. [9] in regions where the SSR markers were tightly linked (generally where they mapped less than 2 cM apart). The exceptions to this trend were loci Ch01d03, which was located at 25 cM on LG12 on the map presented by Fernández-Fernández et al. [9], but which was located 27.3 cM away from its closest marker at the distal end of LG12 in this investigation, and NH014a.z, which was located at 33.3 cM in this investigation on LG17, but was located at the distal end of LG17 on the map of Fernández-Fernández et al. [9], 6 cM from its closest flanking marker. The positions of these markers are presented in red on Figure 3 for comparison, but the markers were removed from the final analysis of the consensus SNP and SSR map presented in Figure 2. The addition of SNP markers to the consensus map reduced the effective comparable length of the M432 SSR map by a total of 74.5 cM (6.1%), but due to the increased coverage of the genome with SNP markers at the ends of LGs where no SSRs had previously been mapped, overall, the linkage map length was increased by 52.7 cM (4.3%). Additional file 1 details the segregating markers mapped in the M432 population including the dbSNP (EMBL) accession code assigned to each on the IRSC array, the LG and map position of each mapped marker, the monogenic marker segregation ratios, associated chi-squared values and predicted pseudo-chromosome positions of each marker on the ‘Golden Delicious’ genome sequence.

### Segregation distortion on the M432 linkage map

A total of six LGs exhibited some level of segregation distortion along their length (Table 1 and Additional file 1). Of these, two (LG1 and LG7) displayed segregation distortion for a short region at their proximal end, whilst the distortion observed on the other four groups (LG3, LG9, LG12 and LG17) was more extensive, the most significant distortion present on LG12 and on LG17 in the region of the *S*-locus.

### Comparison of genetic and physical positions of the IRSC array SNP markers

**Figure 4:**
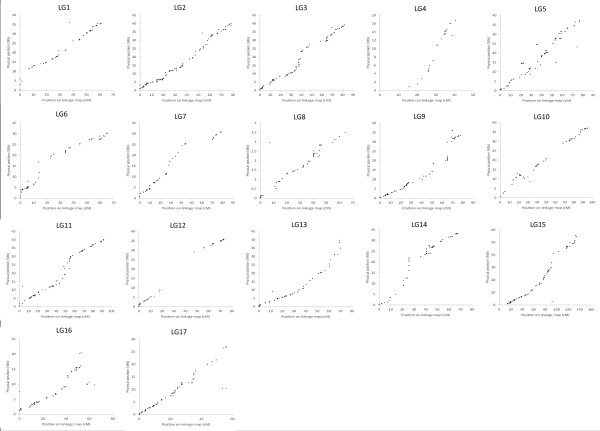
**Comparison of genetic and physical positions of the mapped IRSC SNPs on the M432 genetic and ‘Golden Delicious’ physical maps.** Plots for each of the SNPs mapped in the M432 mapping progeny as a function of their physical positions on the ‘Golden Delicious’ genome sequence. Each plot (LG1-LG17) represents one of the 17 LGs of the M432 map and one of the pseudo-chromosomes (1–17) of the ‘Golden Delicious’ genome sequence.

Figure 4 depicts the physical distances of the mapped SNP markers on the ‘Golden Delicious’ genome sequence plotted against their genetic positions on the M432 map. In general, the within LG genetic positions of the SNP markers that mapped to the M432 LGs were consistent with their positions on the ‘Golden Delicious’ genome sequence and in some cases (LG3, LG6, LG7, LG11 and LG14) distinct domains signifying high and low recombination along the LG/pseudochromosome could be observed . However, each of the 17 LGs contained a small number of markers from more than one ‘Golden Delicious’ pseudo-chromosome, indicating the erroneous placement of genome sequence contigs in the ‘Golden Delicious’ pseudo-chromosomes or the presence of undetected gene paralogues within the *Malus* genome. A total of 311 *Malus* SNP markers mapped to positions on the M432 LGs that did not agree with their physical positions on the ‘Golden Delicious’ genome sequence. The markers were distributed throughout the 17 *Malus* pseudo-chromosomes and represent 13.7% of all markers mapped in the M432 mapping progeny. Marker genetic and physical positions are given in Additional file 1.

## Discussion

We have extended the previously published M432 genetic linkage map using SNP markers generated using the IRSC array, and increased marker saturation on the map from an average of one marker every 3.8 cM to one marker every 0.5 cM. Of the 7,867 *Malus* SNP markers contained on the array, 1,841 (23.4%) were heterozygous in one of the two parents of the progeny, and a further 998 (12.6%) were heterozygous in both parental genotypes. A total of 26 heterozygous SNPs were derived from the 921 *Pyrus* SNPs on the array, indicating a T_SNP_ value of 2.82% transferability to *Malus*, which is within the range of values previously obtained by Micheletti et al. [5] of between 1.2 and 3% transferability of SNPs from *Malus* to *Pyrus*. The total percentage of heterozygous *Malus* SNPs (36%) was within the T_SNP_ value range reported for the transferability of ‘Golden Delicious’ SNPs to rootstock varieties [5], indicating that previously reported T_SNP_ values for *Malus* cultivars should be broadly applicable to the number of SNPs expected to segregate in a given *Malus* genotype on the IRSC array.

### SNP marker heterozygosity and GenomeStudio genotype clustering

Our investigation revealed a large number of SNPs putatively heterozygous and thus segregating in the M432 progeny, but for which genotype calls using GenomeStudio (Illumina) resulted in segregation patterns that could not be assigned a position on one of the 17 LGs of the M432 consensus map. A closer inspection of the genotype data in GenomeStudio (Illumina) revealed low cluster separation calls for all SNPs and a number of different causes for the discrepancies in these data, including many loci for which data were grouped into more than one sub-cluster in each genotype. Sub-clusters are most likely caused by probes detecting more than one locus, one of which contained an SNP or indel, or the detection of more than one allele containing SNPs within the probe sequence [4], leading to non-uniform clustering of the detected genotypes. Such variation has been detected using oligonucleotide arrays in humans, where it is attributed to copy-number variation (CNV) [10]. Sanzol [11] investigated duplicated genes in *Malus* using EST data and concluded that the data supported a model of continuous small-scale duplication events, in addition to the recent whole genome duplication event in the lineage of the genus [1,12]. We suggest that the apparently large number of loci for which segregation data was generated using the IRSC array, but which could not be located to the M432 linkage map (20%) is a result of a combination of factors, including detection of paralogous loci generated through whole genome duplication and CNV, and the mis-assignation of genotypes scored in GenomeStudio (Illumina) due to the lack of a well defined cluster file for the analysis of *Malus*.

### SNP and SSR co-segregation analysis and linkage map construction

To our knowledge, this is the most extensive linkage map of a *Malus* mapping progeny published to date. Previously, the IRSC array had been used to genotype the progeny derived from the cross ‘Royal Gala’ × ‘Granny Smith’ (RG × GS) which was reported by Chagné et al. [7], although in the report, data from only a single LG (LG1) was presented. The number of SNP markers heterozygous in the M432 population was 2,856 (32.5% of the total number of SNPs on the array), almost identical to the 2,810 (32% of the total number of SNPs on the array) reported to be heterozygous in RG × GS by Chagné et al. [7]. Of those markers heterozygous in the M432 progeny, 2,272 markers could be assigned reliable positions on the linkage map presented. Since only one LG was presented in the report of Chagné et al. [7], it was not possible to determine if all 2,810 markers were located to the RG × GS linkage map. Additionally as only markers segregating in a ‘pseudo test-cross’ configuration were presented on the RG × GS LG1 parental linkage maps and the M432 progeny is derived from a back-cross between ‘M.27’ and its semi-vigourous seedling ‘M.116’ (‘M.27’ × ‘M.M.106’), direct comparison between the number of markers mapped in both investigations was not possible. However, the average distance between markers on the RG × GS linkage map was reported to be 0.88 and 0.91 for the ‘Royal Gala’ and ‘Granny Smith’ linkage maps respectively. These distances are comparable to the average genetic distances between the same marker types in this investigation (data not shown) and thus we conclude that, given the diverse pedigrees of these two mapping progenies, the IRSC array should be an invaluable tool for linkage map development and saturation in any given *M. pumila* mapping progeny.

We identified a number of regions of the M432 map where segregation distortion was widespread and extensive. The most extreme example was at the bottom of LG17 where the self-incompatibility is located [13,14]. The M432 progeny was derived from a semi-compatible cross between ‘M.27’ and its semi-vigourous seedling ‘M.116’ (‘M.27’ × ‘M.M.106’), and thus fertilisation could not occur in homozygous genotype combinations around the *S*-locus, leading to the observed segregation distortion. Two other regions of distortion are probably the result of genes associated with the dwarfing phenotype in the cross, since some seedlings of the progeny displayed lethal and sub-lethal dwarf phenotypes. Efforts are on-going to characterise the M432 progeny and identify QTL associated with the dwarfing phenotype as well as a number of other traits including water-use efficiency.

### Genome heterozygosity, SNP marker genome coverage and a comparison of genetic and physical SNP positions

When the genetic positions of the 2,272 *Malus* SNP markers mapped in the M432 progeny were compared to their physical positions on the ‘Golden Delicious’ genome sequence a high degree of colinearity was observed. Additionally, distinct regions of high and low recombination could be observed in a number of cases along the LGs/pseudochromosomes. The anchored portion of the ‘Golden Delicious’ genome from which the physical positions of the SNPs mapped in the M432 progeny were derived covers an estimated 71.2% of the ‘Golden Delicious’ genome and represents the majority of the gene-rich portion of the apple genome sequence [1]. Since the un-sequenced and un-anchored portions of the genome have been predicted to be largely repetitive sequence, and the SNPs positioned on the IRSC array were derived almost exclusively from exonic sequence [7], it was expected that plots of genetic and physical positions of the SNPs mapped in the M432 progeny would reveal a largely linear relationship for each chromosome due to the absence of markers mapped to the largely repetitive centromeric and telomeric regions of the chromosomes. However, many of the plots revealed non-linear relationships, with clear regions of high recombination representing chromosome arms, and centres of low recombination representing putative centromeric regions, as have been observed in similar plots of other organisms such as *Caenorhabditis elegans*[15] and *Oryza sativa*[16]. Regions of effectively zero recombination at the LG ends which would indicate the presence of telomeric regions were largely undetected in this investigation. However, at least one region of zero recombination was observed on plots of LG/pseudochromosomes LG1, LG6, LG7 and LG12.

A total of 311 SNP markers (13.7% of all mapped SNP markers) were located to positions on LGs that conflicted with the predicted positions of the SNPs on the ‘Golden Delicious’ pseudo-chromosomes [1]. Upon closer inspection of the predicted positions of these SNP markers, 47 (15%) mapped to regions in the M432 mapping progeny that were homeologous to those predicted, whilst the remaining 264 (85%) were located in non-homeologous regions of the genome, indicating the presence of paralogous genomic regions, or possible mis-assignment of genome sequence contigs during the assembly and anchoring of the ‘Golden Delicious’ genome sequence.

Human and animal genetics employ genetic information gained from individuals who are members of extended pedigrees for the identification of loci controlling complex genetic traits [17,18]. One of the aims of the IRSC was to produce a genotyping tool to enable the determination of SNP haplotypes for apple varieties related through well-defined pedigrees [7], leading to the identification of genes controlling important agronomic traits using a pedigree-based analysis. The advantages of such analyses are that the full variation associated with a trait of interest can be sampled within a gene pool; however, it is essential that markers included in each haplotype are derived from physically associated locations on the *Malus* genome. If the 13.7% of the markers mapped in the M432 progeny in this investigation that were identified as misplaced on the ‘Golden Delicious’ genome sequence of Velasco et al. [1], had been used to create haplotypes for pedigree-based association studies, they would have exhibited recombination at higher frequencies than linked markers within the haplotype, making genetic data within the pedigree difficult or impossible to interpret. In total, 2,272 (25.8%) of the SNP markers contained on the IRSC array were mapped in the M432 progeny. If the markers mapped in the M432 progeny are representative of all SNPs contained on the array, there are potentially a further 767 SNPs for which the genetic and predicted genomic positions may disagree, potentially leading to the creation of false haplotypes for *Malus* cultivars.

## Conclusions

We have employed the IRSC *Malus* array to extend and saturate the SSR linkage map of the M432 mapping progeny. The use of the array enabled us to locate a total of 2,270 SNPs to the consensus linkage map at a fraction of the time and cost of developing a similar map using other experimental approaches, and to develop the most comprehensive saturated complete linkage map for a *Malus* mapping progeny to date. The mapping of the SNPs enabled us to assess the relative coverage and accuracy of the ‘Golden Delicious’ genome sequence and to identify a significant proportion (13.7%) of SNPs that had been erroneously located to the ‘Golden Delicious’ pseudochromosomes. Further mapping in additional progenies will help to characterise regions of the ‘Golden Delicious’ sequence that are incorrectly placed in the current assembly version, and assist in the continued improvement of the reference sequence for *Malus*.

## Methods

### Plant material and DNA extraction

The M432 rootstock progeny used in this investigation was first described by Evans et al. [8]. It was derived from the cross ‘M.27’ × ‘M.116’ (‘M.M.106’ × ‘M.27’) and comprises 140 seedlings. DNA from the seedlings of the M432 progeny was used to generate SNP data using the IRSC array, and subsequently to construct SNP-based linkage maps of the parental genomes. DNA was freshly isolated from young leaf tissue of ‘M.27’, ‘M.116’ and the 140 seedlings of the M432 progeny using the DNeasy plant miniprep kit (Qiagen) according to the manufacturer’s protocol immediately prior to genotyping. It was then quantified using PicoGreen (Invitrogen) against a λ standard DNA dilution series using a Synergy 2 fluorimeter (BioTek). DNA from all seedlings was diluted to 50 ng/ul for genotyping using the IRSC array.

### Malus Infinium® II whole genome genotyping array

The IRSC array, employing exclusively Illumina Infinium® II design probes and dual colour channel assays (Infinium HD Assay Ultra, Illumina), that was described previously by Chagné et al. [7] was used for genotyping the progeny of the M432 apple mapping population. The IRSC bead chips contained a total of 7,867 possible *Malus*, and 921 possible *Pyrus* SNPs. Progeny DNA was assayed following the manufacturer’s recommendations. Briefly, a whole genome amplification reaction was performed, followed by denaturation and hybridization to the IRSC BeadChips (Illumina). Un-hybridized and non-specifically hybridized DNA was removed through washing, following which a single base extension reaction was performed to incorporate differentially labeled nucleotides at the SNP sites of each of the 8,788 probes of the BeadChip for each M432 genotype. BeadChips were then imaged and data were collected using the HiScan detection platform (Illumina) following Illumina-published standard operating procedures (http://www.illumina.com).

### Data scoring and SNP nomenclature

Data generated for the 8,788 SNPs in the 140 seedlings and the two parents of the M432 progeny were scored using (Illumina) using a GenCall threshold of 0.15. Data were then exported into (Microsoft Inc.) for post scoring processing before data analysis. Prior to linkage analysis markers with a GenTrain score below 0.4 were excluded from the dataset. Following initial linkage analyses, all markers not mapping to one of the expected 17 LGs were visually inspected for accuracy, to determine data quality, and to resolve any errors created by automatic allele calling in (Illumina). Data for all SNPs showing putative segregation were discarded prior to mapping if one or both of the parental genotypes failed to amplify in the assay or if segregation data contained genotypes not expected from the parental genotypes.

### Data analysis and M432 linkage map construction

Markers were re-coded using genotype codes for linkage analysis using (Kyasma, Wageningen, NL), according to their segregation type; AB × AA or AB × BB (segregating in the female ‘M.27’ genotype) were coded   and AA × AB or BB × AB (segregating in the male ‘M.116’ genotype) were coded   . Markers segregating AB × AB (in both parental genotyes) were recoded   . SNP nomenclature in this investigation followed the dbSNP (EMBL) accession codes for each SNP on the array that has been deposited in the Genome Database for Rosaceae [19].

Linkage mapping was performed with all SNP data and the SSR data of Fernández-Fernández et al. [9]. Data for SSR markers with parental genotypes   (and thus segregating in a 3:1 Mendelian ratio) were not considered in this analysis. Segregation data for SNPs were analysed and consensus genetic linkage maps were obtained using a two-step procedure. Initially SNP markers were ordered using the QMAP procedure of Genstat 14 [20]. For outbreeders QMAP employs the method of Jansen [21] for ordering markers, in combination with the EM algorithm using a hidden Markov model for obtaining multipoint maximum likelihood estimates of recombination frequencies [22]. SNP marker orders were then fixed and SSRs were added to the consensus SNP linkage map using (Kyazma, NL); marker placement was determined using a minimum LOD score threshold of 3.0, a recombination fraction threshold of 0.35, ripple value of 1.0, jump threshold of 3.0 and a triplet threshold of 5.0, and mapping distances were calculated using the Kosambi mapping function. Linked markers were only considered as constituting a LG if more than six markers coalesced into a single group. Any markers that remained ‘unlinked’ following co-segregation analysis or that were contained in groups of fewer than six markers were visually inspected in (Illumina). All linkage maps presented were plotted using for Windows [23] and LG nomenclature for M432 follows the numbering reported previously for this progeny by Fernández-Fernández et al. [9].

### Comparison of genetic locations and physical positions on the ‘Golden Delicious’ genome sequence

Physical positions of all SNP markers were derived from their predicted positions on the ‘Golden Delicious’ genome sequence and were plotted as a function of genetic distances on the M432 consensus map. Mareymaps for each individual chromosome were produced using (Microsoft Inc.).

## Competing interests

The authors declare that they have no competing financial interests.

## Authors’ contributions

LA carried out the experiments, analysed the data and co-authored the manuscript. FFF conceived the experiments, managed the populations and co-authored the manuscript. JJ analysed the data. EB carried out the experiments. KME conceived the experiments and critically evaluated the manuscript. RVio advised on the experiments. RVel managed the experiments. JMD advised on the experiments and critically evaluated the manuscript. MT conceived the experiments, managed the experiments, analysed the data and co-authored the manuscript. DJS conceived the experiments, managed the experiments, analysed the data and authored the manuscript. All authors read and approved the final manuscript

## Supplementary Material

Additional file 1 **Segregation data for the 2,579 markers segregating in the M432 progeny.** The 2,272 segregating IRSC array SNP markers mapped in the M432 population along with the 307 previously mapped SSR and *S*-locus markers, including the SNP name, the dbSNP (EMBL) accession code assigned to each on the IRSC array, the LG and map position of each mapped marker, the monogenic marker segregation ratios, associated chi-squared values and the predicted pseudo-chromosome positions of each marker on the ‘Golden Delicious’ genome sequence. Markers for which genetic and physical positions conflicted are highlighted in red. Click here for file
